# Elemental characterization of oral cavity squamous cell carcinoma and its relationship with smoking, prognosis and survival

**DOI:** 10.1038/s41598-020-67270-5

**Published:** 2020-06-25

**Authors:** Anderson Barros Archanjo, Arícia Leone Evangelista Monteiro de Assis, Mayara Mota de Oliveira, Suzanny Oliveira Mendes, Aline Ribeiro Borçoi, Lucas de Lima Maia, Rafael Pereira de Souza, Rafael de Cicco, Kelly Cristina Saito, Edna Teruko Kimura, Marcos Brasilino de Carvalho, Fabio Daumas Nunes, Eloiza H. Tajara, Marcelo dos Santos, Breno Valentim Nogueira, Leonardo Oliveira Trivilin, Christiano Jorge Gomes Pinheiro, Adriana Madeira Álvares-da-Silva

**Affiliations:** 1 Postgraduate Program in Biotechnology/RENORBIO, Federal University of Espirito Santo, Av. Marechal Campos, 1468, Vitoria, 29.040-090 ES Brazil; 2 Cancer Institute Arnaldo Vieira de Carvalho, São Paulo, Brazil; 3 Institute of Biomedical Science, University of São Paulo, São Paulo, Brazil; 4 Molecular Biology Laboratory, Heliópolis Hospital, São Paulo, Brazil; 5 School of Dentistry, University of São Paulo, São Paulo, Brazil; 6 Medical School of São José do Rio Preto, São José do Rio Preto, Brazil; 7 Multicampi School of Medical Sciences of Rio Grande do Norte, Federal University of Rio Grande do Norte, Caicó, Brazil; 8 Center for Agricultural Sciences and Engineering, Federal University of Espirito Santo, Alegre, Brazil

**Keywords:** Biomarkers, Fluorescence spectroscopy

## Abstract

Oral cancer squamous cell carcinoma (OCSCC) mainly affects individuals aged between 50 and 70 years who consume tobacco and alcohol. Tobacco smoke contains hundreds of known toxic and carcinogenic molecules, and a few studies have sought to verify the relationship of such trace elements as risk or prognostic factors for head and neck cancer. We obtained 78 samples of tumor tissues from patients with OCSCC, and performed a qualitative elemental characterization using the micro X-Ray Fluorescence technique based on synchrotron radiation. We found the presence of magnesium, phosphorus, sulfur, chlorine, potassium, calcium, chromium, manganese, iron, zinc, cobalt, nickel, copper, arsenic and bromine in OCSCC samples. Magnesium, chlorine, chromium, manganese, nickel, arsenic and bromine are associated with smoking. We observed a significant association between relapse and chlorine and chromium. The presence of chlorine in the samples was an independent protective factor against relapse (OR = 0.105, CI = 0.01–0.63) and for best disease-free survival (HR = 0.194, CI = 0.04–0.87). Reporting for the first time in oral cancer, these results suggest a key relationship between smoking and the presence of certain elements. In addition, chlorine proved to be important in the context of patient prognosis and survival.

## Introduction

Squamous cell carcinoma (SCC) accounts for more than 90% of oral cancer cases^1,2^ and mainly affects individuals aged between 50 and 70 years who consume tobacco and alcohol. It may also be associated with human papillomavirus infection, genetic susceptibility and passive smoking exposure^1–5^. Tobacco smoke itself, which contains hundreds of known toxic and carcinogenic molecules^6,7^, can induce DNA damage^8^. Among these substances are several inorganic compounds, such as arsenic, calcium, chlorine, cobalt, copper, chromium, iron, magnesium, manganese, nickel, potassium, zinc and others^9,10^.

The analysis of trace elements is quite uncommon in the head and neck cancer field. Over the past two decades, a few studies have sought to verify the identity of these and other trace elements as risk or prognostic factors for head and neck cancer. Some authors verified the differences in the profile of trace elements in the blood (plasma/serum) of patients with or without head and neck cancer^11–19^. Differences were also found in the analyses performed on the hair and nails of patients with head and neck cancer^15,17,20,21^. However, in studies carried out on saliva, there were divergent results. Dziewuska *et al*.^22^ found no evidence of mineral markers in oral cancer, whereas Shetty *et al*.^23^ reported an increase in copper and a decrease in zinc in the saliva of patients with oral cancer.

A few studies have been conducted on tumor tissues. In thyroid cancer, for example, Zaichick *et al*.^24^ found higher levels of silver, cobalt, mercury, iodine and rubidium in malignant nodules and Baltaci *et al*.^25^ found changes in the levels of zinc and selenium in thyroid tissues. Regarding breast cancer, Majewska *et al*.^26^ reported differences in the concentration of some elements between benign and malignant tumors. Geraki *et al*.^27^ found that an increase in potassium and zinc is bigger than that of iron and copper. Silva *et al*.^28^ reported that trace elements may be potential markers in breast tumors and the authors emphasized that patients with elemental copper presence had lower survival.

In view of the above, most of the studies carried out used other types of samples, such as blood, hair, nails and saliva, and the studies that used tumor tissue were from anatomical sites different from ours. Thus, there is no consensus on what elements could be found in head and neck cancer. Given the knowledge gap in the link between tobacco elements and oral cancer prognosis, this study aimed to qualitative characterize the presence of elements in oral cavity cancers samples according to smoking status and verify the possible association of these elements with prognosis and survival.

## Results

### Relationship between smoking status and elemental characterization

The elements found in oral cavity SCC (OCSCC) samples were magnesium, phosphorus, sulfur, chlorine, potassium, calcium, chromium, manganese, iron, zinc, cobalt, nickel, copper, arsenic and bromine (Table 1).

**Table 1:** Elemental characterization of oral cavity squamous cell carcinoma samples according to patients’ smoking habits.

Element	All patients (n = 78)	Smoking				History of tobacco consumption			Current tobacco consumption		
		Never (n = 14)	Former (n = 23)	Current (n = 41)	*P-*	No^1^ (n = 14)	Yes^2^ (n = 64)	*P-*	No^3^ (n = 37)	Yes^4^ (n = 41)	*P-*
**Magnesium**											
Absent	69 (88.5)	14 (100.0)	15 (65.2)	40 (97.6)	***0.000***	12 (85.7)	57 (89.1)	0.722	31 (83.8)	38 (92.7)	0.191
Present	9 (11.5)	0 (0.0)	8 (34.8)	1 (2.4)		2 (14.3)	7 (10.9)		6 (16.2)	3 (7.3)	
**Phosphorus**											
Absent	5 (6.5)	1 (7.1)	1 (4.4)	3 (7.3)	0.891	2 (14.3)	3 (4.7)	0.184	2 (5.4)	3 (7.3)	0.549
Present	73 (93.6)	13 92.9)	22 (95.6)	38 (92.7)		12 (85.7)	61 (95.3)		35 (94.6)	38 (92.7)	
**Sulfur**											
Absent	1 (1.3)	0 (0.0)	1 (4.4)	0 (0.0)	0.298	0 (0.0)	1 (1.5)	0.638	1 (2.7)	0 (0.0)	0.474
Present	77 (98.7)	14 (100.0)	22 (95.6)	41 (100.0)		14 (100.0)	63 (98.5)		36 (97.3)	41 (100.0)	
**Chlorine**											
Absent	50 (64.1)	12 (85.7)	14 (60.9)	24 (58.5)	0.174	11 (78.6)	39 (60.9)	0.213	28 (75.7)	22 (53.6)	***0.043***
Present	28 (35.9)	2 (14.3)	9 (39.1)	17 (41.5)		3 (21.4)	28 (39.1)		9 (24.3)	19 (46.4)	
**Potassium**											
Absent	14 (17.9)	4 (28.6)	3 (13.1)	7 (17.1)	0.480	5 (35.7)	9 (14.1)	0.056	8 (21.6)	6 (14.6)	0.422
Present	64 (82.1)	10 (71.4)	20 (86.9)	34 (82.9)		9 (64.3)	55 (85.90		29 (78.4)	35 (85.4)	
**Calcium**											
Absent	1 (1.3)	1 (7.1)	0 (0.0)	0 (0.0)	0.099	1 (7.1)	0 (0.0)	0.179	1 (2.7)	0 (0.0)	0.474
Present	77 (98.7)	13 (92.90	23 (100.0)	41 (100.0)		13 (92.8)	64 (100.00		36 (97.3)	41 (100.0)	
**Chromium**											
Absent	45 (57.7)	13 (92.9)	9 (39.1)	23 (56.1)	***0.006***	13 (92.9)	32 (50.0)	***0.003***	24 (64.9)	21 (51.2)	0.223
Present	33 (42.3)	1 (7.1)	14 (60.9)	18 (43.9)		1 (7.10)	32 (50.0)		13 (35.1)	20 (48.7)	
**Manganese**											
Absent	52 (66.7)	14 (100.0)	10 (43.5)	28 (68.3)	***0.002***	12 (85.7)	40 (66.7)	0.095	26 (70.3)	26 (63.4)	0.521
Present	26 (33.3)	0 (0.0)	13 (56.5)	13 (31.7)		2 (14.3)	26 (33.3)		11 (29.7)	15 (36.6)	
**Iron**											
Absent	0 (0.0)	0 (0.0)	0 (0.0)	0 (0.0)	1.000	0 (0.0)	0 (0.0)	1.000	0 (0.0)	0 (0.0)	1.000
Present	78 (100.0)	14 (100.0)	23 (100.0)	41 (100.0)		14 (100.0)	64 (100.0)		37 (100.0)	41 (100.0)	
**Cobalt**											
Absent	69 (88.5)	12 (85.7)	18 (78.3)	39 (95.1)	0.121	10 (71.4)	59 (92.2)	0.050	32 (86.5)	37 (88.5)	0.433
Present	9 (11.5)	2 (14.3)	5 (21.7)	2 (4.9)		4 (28.6)	5 (7.8)		5 (13.5)	4 (11.5)	
**Nickel**											
Absent	67 (85.9)	14 (100.0)	20 (86.9)	33 (80.5)	0.191	13 (92.8)	54 (84.4)	0.409	36 (97.3)	31 (75.6)	***0.005***
Present	11 (14.1)	0 (0.0)	3 (13.1)	8 (19.5)		1 (7.2)	10 (15.6)		1 (2.7)	10 (24.4)	
**Copper**											
Absent	4 (5.1)	1 (7.1)	1 (4.3)	2 (4.9)	0.927	1 (7.1)	3 (4.7)	0.706	2 (5.4)	2 (4.9)	0.652
Present	74 (94.9)	13 (92.9)	22 (95.7)	39 (95.1)		13 (92.9)	61 (95.3)		35 (94.6)	39 (95.1)	
**Zinc**											
Absent	0 (0.0)	0 (0.0)	0 (0.0)	0 (0.0)	1.000	0 (0.0)	0 (0.0)	1.000	0 (0.0)	0 (0.0)	1.000
Present	78 (100.0)	14 (100.0)	23 (100.0)	41 (100.0)		14 (100.0)	64 (100.0)		37 (100.0)	41 (100.0)	
**Arsenic**											
Absent	25 (32.1)	9 (64.3)	4 (17.4)	12 (29.3)	***0.011***	8 (57.1)	17 (26.5)	**0.026**	14 (37.8)	11 (26.9)	0.298
Present	53 (67.9)	5 (35.7)	19 (82.6)	29 (70.7)		6 (42.9)	47 (73.5)		23 (62.2)	30 (73.1)	
**Bromine**											
Absent	25 (32.1)	9 (64.3)	4 (17.4)	12 (29.3)	***0.011***	8 (57.1)	17 (26.5)	***0.026***	14 (37.8)	11 (26.9)	0.298
Present	53 (67.9)	5 (35.7)	19 (82.6)	29 (70.7)		6 (42.9)	47 (73.5)		23 (62.2)	30 (73.1)	

^1^No, Never; ^2^Yes, Former/Current; ^3^No, Never/Former; ^4^Yes: Current; Data represented as absolute counts (%).

Stratification of the elements by smoking status revealed that the presence of magnesium (p < 0.0001), chromium (p = 0.006) and manganese (p = 0.002) was associated with smoking. The highest percentages of these elements were found in individuals who had stopped smoking, whereas magnesium and manganese were absent in those who had never smoked. Arsenic and bromine (p = 0.011 for both) were present in the highest amounts in individuals who had a smoking habit, whether in the past or present (Table 1).

Chromium was associated with a history of tobacco use (p = 0.003), whereas it was absent in the cancer tissue samples of individuals who had never used tobacco. Arsenic and bromine (p = 0.026 for both) were present in the highest amounts in individuals with a history of tobacco consumption (Table 1).

Regarding current tobacco consumption, elemental stratification showed an association between chlorine and current tobacco consumption (p = 0.043). Only 25% of individuals who currently consumed tobacco had detectable chlorine in the tumor samples. Nickel, on the other hand, was present in less than 3% of the tumor samples of patients who did not currently consume tobacco (p = 0.005) (Table 1).

### Role of elements as prognostic factors in oral cancer

**Figure 1:**
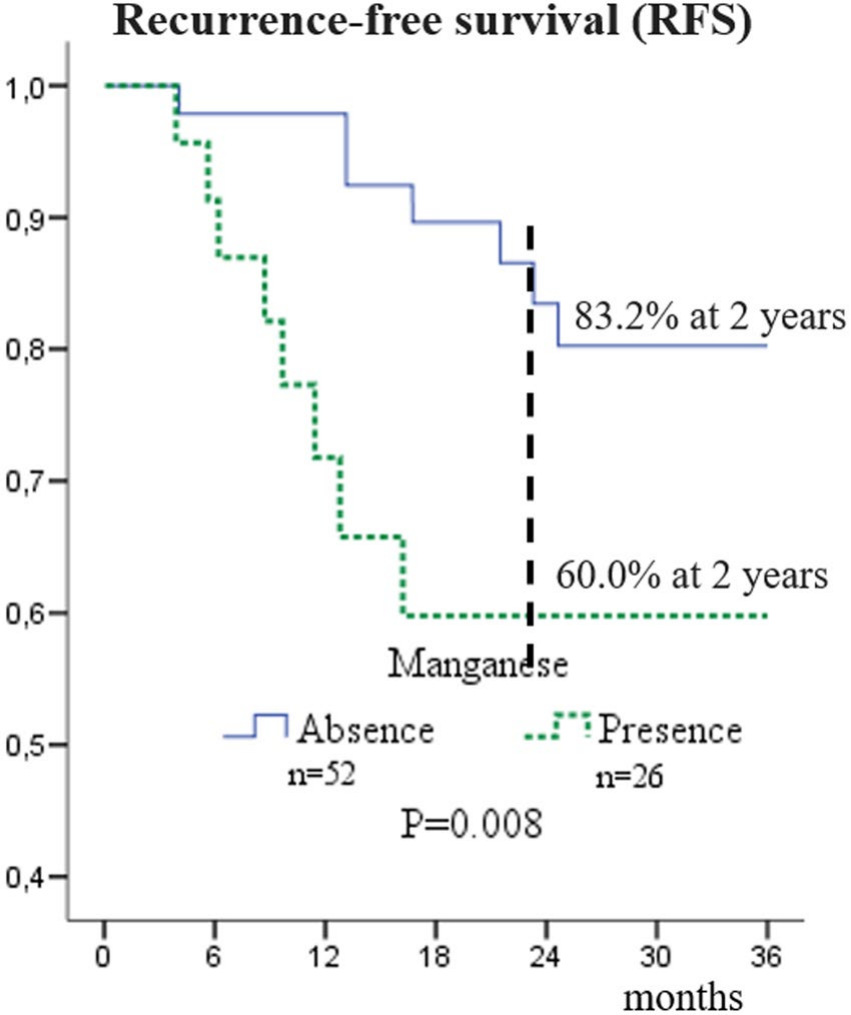
Recurrence-free survival (RFS) curves of patients with squamous cell carcinoma of the oral cavity according to status of manganese.

We identified associations between the presence of some elements and the prognosis of patients with oral cancer. There was a significant association between manganese and recurrence-free survival (Wilcoxon p = 0.008). During the first 12 months after surgery, 40.0% of patients with manganese present in oral cavity cancer samples suffered from recurrence while only 16.8% of patients without manganese detection had relapsed (see Fig. 1).

**Figure 2:**
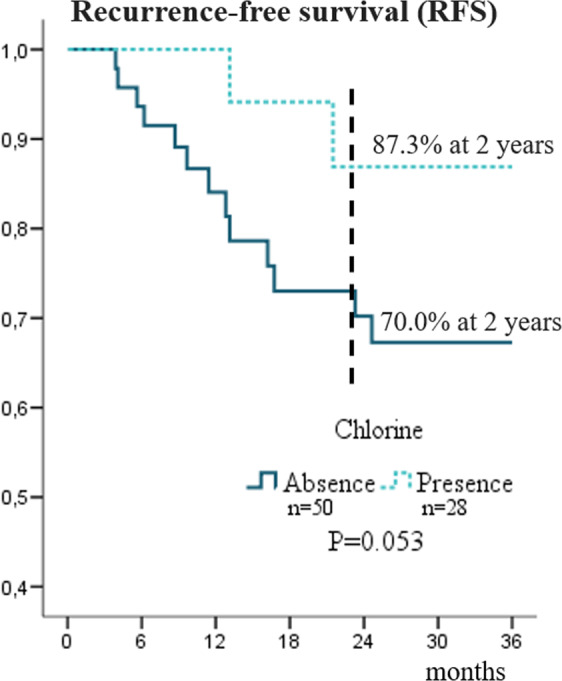
Recurrence-free survival (RFS) curves of patients with squamous cell carcinoma of the oral cavity according to status of chlorine.

We observed a significant association between relapse and chlorine and chromium (p = 0.037 and p = 0.034, respectively; Table 2). In a multivariate analysis, the presence of chlorine in the samples was an independent protective factor against relapse, with a reduced risk (odds ratio [OR] = 0.105, confidence interval [CI] = 0.01–0.63; Table 3). Regarding chlorine, we also found that at 18 months after surgery, 30% of patients without chlorine in their samples had relapsed, whereas in the same period only 12.8% of the patients with chlorine suffered from recurrence (see Fig. 2). Multivariate analysis revealed that the presence of chlorine was an independent protective factor for better disease-free survival, conferring a reduced risk (hazard ratio [HR] = 0.194, CI = 0.04–0.87; Table 3).

**Table 2:** Elemental characterization in association recurrence and death.

Element	Recurrence			Death		
	No (n = 63)	Yes (n = 15)	*P-*	No (n = 49)	Yes (n = 29)	*P-*
**Magnesium**						
Absent	54 (85.7)	15 (100.0)	0.129	44 (89.8)	25 (86.2)	0.446
Present	9 (14.3)	0 (0.0)		5 (10.2)	4 (13.8)	
**Phosphorus**						
Absent	4 (6.3)	1 (6.7)	0.620	5 (10.2)	0 (0.0)	0.090
Present	59 (93.7)	14 (93.3)		44 (89.8)	29 (100.0)	
**Sulfur**						
Absent	1 (1.6)	0 (0.0)	0.807	1 (2.0)	0 (0.0)	0.628
Present	62 (98.4)	15 (100.0)		48 (98.0)	29 (100.0)	
**Chlorine**						
Absent	37 (58.7)	13 (86.7)	***0.037***	31 (63.3)	19 (65.5)	0.841
Present	26 (41.3)	2 (13.3)		18 (36.7)	10 (34.5)	
**Potassium**						
Absent	11 (17.5)	3 (20.0)	0.532	8 (16.3)	6 (20.7)	0.627
Present	52 (82.5)	12 (80.0)		41 (83.7)	23 (79.3)	
**Calcium**						
Absent	1 (1.6)	0 (0.0)	0.807	1 (2.0)	0 (0.0)	0.628
Present	62 (98.4)	15 (100.0)		48 (98.0)	29 (100.0)	
**Chromium**						
Absent	40 (63.5)	5 (33.3)	***0.034***	39 (59.2)	16 (55.2)	0.729
Present	23 (36.5)	10 (66.7)		20 (40.8)	13 (44.8)	
**Manganese**						
Absent	45 (71.4)	7 (46.7)	0.067	35 (71.4)	17 (58.6)	0.246
Present	18 (28.6)	8 (53.3)		14 (28.6)	12 (41.4)	
**Iron**						
Absent	0 (0.0)	0 (0.0)	1.000	0 (0.0)	0 (0.0)	1.000
Present	63 (100.0)	15 (100.0)		49 (100.0)	29 (100.0)	
**Cobalt**						
Absent	54 (85.7)	15 (100.0)	0.129	43 (87.8)	26 (89.7)	0.554
Present	9 (14.3)	0 (0.0)		6 (12.2)	3 (10.3)	
**Nickel**						
Absent	54 (85.7)	13 (86.7)	0.645	41 (83.7)	26 (89.7)	0.353
Present	9 (14.3)	2 (13.3)		8 (16.3)	3 (10.3)	
**Copper**						
Absent	3 (4.8)	1 (6.7)	0.561	4 (8.2)	0 (0.0)	0.149
Present	60 (95.2)	14 (93.3)		44 (91.8)	29 (100.0)	
**Zinc**						
Absent	0 (0.0)	0 (0.0)	1.000	0 (0.0)	0 (0.0)	1.000
Present	63 (100.0)	15 (100.0)		49 (100.0)	29 (100.0)	
**Arsenic**						
Absent	18 (28.6)	7 (46.7)	0.177	16 (32.7)	9 (31.0)	0.544
Present	45 (71.4)	8 (53.3)		33 (67.3)	20 (69.0)	
**Bromine**						
Absent	18 (28.6)	7 (46.7)	0.177	16 (32.7)	9 (31.0)	0.544
Present	45 (71.4)	8 (53.3)		33 (67.3)	20 (69.0)	

Data represented as absolute counts (%).

**Table 3:** Multivariable logistic regression model and Cox model of prognostic factors and survival in patients which oral squamous cell carcinoma.

	Logistic regression model				Cox model			
	Recurrence		Death		Recurrence-free survival		Overall survival	
	OR (CI 95%)	P-	OR (CI 95%)	P-	HR (CI 95%)	P-	HR (CI 95%)	P-
**N (TNM)**^**1**^								
Negative	1		1		1		1	
Positive	4.912 (1.20–20.06)	**0.027**	5.614 (1.93–16.26)	***0.001***	3.081 (1.38–6.85)	***0.006***	3.081 (1.38–6.85)	***0.006***
**Sex**								
Female	1							
Male	9.095 (0.95–87.10)	0.055						
**Age**								
≤ 63 years	1				1			
> 63 years	0.194 (0.04–0.93)	***0.040***			0.264 (0.07–0.95)	***0.042***		
**CAC**								
No^2^			1				1	
Yes^3^			2.844 (1.00–8.01)	***0.048***			1.804 (0.80–4.06)	0.154
**Chlorine**								
Absence	1				1			
Present	0.105 (0.01–0.63)	***0.014***			0.194 (0.04–0.87)	***0.033***		
**Manganese**								
Absence							1	
Present							1.751 (0.81–3.75)	0.151

OR, Odds Ratio; HR, Hazard Ratio; CAC, Current alcohol consumption; CI, confidence interval; ^1^TNM classification 7th edition; ^2^No, Never/Former; ^3^Yes, Current.

## Discussion

Determining the elemental profile of head and neck cancer tumor tissue is a very novel and highly relevant issue, as it may reveal not only the relationship between these elements and smoking, but also the influence of such elements on the prognosis and survival of cancer patients. To the best of our knowledge, this is the first study to perform a qualitative elemental characterization (presence or absence) of tumor tissue samples from patients with OCSCC using the micro-XRF technique based on synchrotron radiation and correlate these elements with smoking habits, prognosis and survival. In our study, we verified the presence of the elements magnesium, phosphorus, sulfur, chlorine, potassium, calcium, chromium, manganese, iron, zinc, cobalt, nickel, copper, arsenic and bromine, of which some were associated with smoking, prognosis, and survival of our patients. A similar approach was performed in patients with thyroid cancer^24,25^ and breast cancer^26–28^. Other studies in head and neck cancer patients have analyzed the levels of some trace elements in the blood^11–19^, hair or nails^15,17,20,21^ and in saliva^22,23^. It is worth mentioning that we found few studies that analyzed trace elements in the tumor tissue, these being in thyroid or breast cancer.

We found that magnesium, chlorine, chromium, manganese, nickel, arsenic and bromine were associated with smoking habits, be it a former or current habit. This link is important because cigarette smoke contains innumerable chemical substances and compounds, some of which are the elements found in tumor tissue samples in this study. Tobacco smoke is a complex and reactive mixture containing more than 5,000 chemicals^29^, including elements, such as magnesium, potassium, calcium, chromium, manganese, iron, cobalt, nickel, copper and zinc, semimetal arsenic, non-metal chlorine, and others^7,9,10^. In addition, studies show that even smokeless tobacco products contribute to the accumulation of nickel and arsenic and that can be synergistic to the risk factors associated with oral cancer^30,31^.

In our study, we verified the presence of other elements, such as zinc and copper, which, despite not having a significant association with smoking, were present in the tumor samples, and thus deserve to be highlighted. The literature shows us that such elements, even in tissue, nails, hair or blood, can be associated with smoking and pose a risk for head and neck cancer. Studies have also shown that low levels of zinc in the nails pose a high risk for oral cancer, even when adjusted for smoking and alcohol use^19,32^. In their study, Wozniak *et al*.^21^ analyzed the hair of patients with laryngeal cancers and found lower levels of zinc in smokers than in nonsmokers. This may be due to the cadmium contained in cigarette smoke having an antagonistic effect on zinc, leading to zinc reduction^33^. In blood, for example, Bandeira *et al*.^18^ reported lower copper concentrations in patients with oral cancer, although these were nonsmokers. However, Chen *et al*.^19^ demonstrated the relationship between copper and zinc in the context of smoking; they observed higher concentrations of copper in smokers, which conferred a greater risk for oral cancer. Thus, it is worth mentioning that zinc and copper are important elements in head and neck cancer, and that their role in tumor tissues should be studied further.

In addition to the relationships already demonstrated with smoking, chromium and manganese were associated with recurrence and recurrence-free survival, respectively. Khlifi *et al*.^34^ reported higher concentrations of chromium and nickel in the blood of patients with head and neck cancer, with concentrations being the highest in smokers. Yuan *et al*.^35^ found an association between serum levels of nickel and chromium and oral cancer. The same authors reported that the concentrations of chromium and nickel were higher in smokers. In addition to tobacco, other sources of exposure can lead to contamination with chromium. In this context, Tsai *et al*.^36^ found higher levels of chromium in patients with oral cancer that was associated with possible soil contamination in Taiwan. Some studies report that chromium in low concentrations can be used for medicinal purposes, and that it is involved in the metabolism of carbohydrates, lipids and proteins. Nevertheless, in sufficiently high concentrations, chromium becomes toxic and carcinogenic^37^. However, none of the authors cited analyzed the relationship of chromium with prognostic and survival factors. Thus, further studies should be carried out to verify the real relationship of chromium with these factors, with this study being an initial source of observation. Nevertheless, studies point to consistent evidence that patients who maintain smoking habits after being diagnosed with head and neck cancer have the lowest survival rates and the highest rates of recurrence^38^. Moreover, tobacco and alcohol consumption influence overall survival, highlighting the importance of discontinuing the use of these substances^39^. In addition, smoking, alcohol consumption, and an inadequate diet can disrupt the levels of elements necessary for the body to function properly and increase the concentrations of toxic and health-threatening metals^40^.

The element chlorine was identified as a protective factor for recurrence and for better recurrence-free survival. Chloride ions (CI^−^) have great physiological relevance with an important role in cellular homeostasis under physiological and pathological conditions. Variations in Cl^−^ flow are associated with cell volume regulation, secretory processes and cellular pH maintenance, all of which are essential to maintain enzymatic activity and cell cycle^41–44^. Recent studies have shown that Cl^−^ act as secondary messengers; their concentration within the cell is dynamic, modulating the activity of transferrin, glucose-6-phosphatase and hemoglobin, among others^45^. The role of Cl^−^ in cellular physiology is well defined; nevertheless, their relationship with cancer pathogenesis remains unclear. Following the discovery of binding of multidrug resistance protein (MDR/P-glycoprotein) to the activity of volume-activated chloride channels in the cancer cells of patients undergoing chemotherapy, chlorine channels gained prominence^46^. Some studies have reported a relationship between chlorine channel expression and patient prognosis and survival. Ruiz *et al*.^47^ found that expression of ANO1 (Ca^2+^-activated chloride channel) causes cell migration, leading to a poor prognosis of head and neck squamous cell carcinoma. Duvvuri *et al*.^48^ reported that expression of TMEM16A (also known as TAOS2, DOG1, and ANO1) induced proliferation and activation of ERK1/2 and was associated with decreased survival. Britschgi *et al*.^49^ demonstrated that calcium-activated chloride channels (ANO1) promoted cancer progression in tumors, such as breast cancer.

In this study, the presence of chlorine as a protective factor for recurrence and better disease-free survival can be explained by several factors: 1) alteration in intracellular pH—tumor cells reside in an acidic environment and intracellular processes compensate for conditions to maintain viability and proliferation;^50^ 2) changes in the actin cytoskeleton—several members of the chloride channel (CLC) family bind to actin and this binding is important for regulation of Cl^−^ transport by these proteins;^51^ 3) cell cycle interference—chloride channel activity of the CLC and CLIC (Chloride intracellular channel) families were associated with changes in cell cycle, and CLC2 is regulated by cdc2/cyclinB, suggesting a mechanism for M phase activation;^52^ CLIC-4 is located in the centrosome and middle of the M-phase cells and CLIC-1 is expressed on the plasma membrane during M phase in CHO-K1 cells^53^.

In conclusion, this study, for the first time, demonstrates the presence of important trace elements and non-essential or toxic elements in samples of OCSCC. Tobacco use predisposes patients to the accumulation of potentially toxic and/or carcinogenic elements. Both manganese and chlorine elements proved to be important prognostic and survival factors for patients with head and neck cancer. Furthermore, as there is no consensus on which elements, beyond the physiological ones, that could be found in head and neck cancer, the present study can serve as a basis for new quantitative studies, where efforts would be directed towards the analysis of the levels of such elements, which would save time and resources for researchers, since these analyzes are costly.

## Materials and Methods

### Samples

Samples were collected by the Head and Neck Genome Project (GENCAPO). For this study, we obtained 78 samples of tumor tissues from 78 patients diagnosed with OCSCC. In order to avoid possible of the samples with paraffin, xylene and alcohol, the quality and integrity of these reagents were checked for purity in each batch used. Diagnosis of OCSCC was confirmed by two pathologists and the patients were treated surgically at the Department of Head and Neck Surgery at the Cancer Institute Arnaldo Vieira de Carvalho (ICAVC), São Paulo, Brazil, from January 2012 to May 2015. The follow-up was at least 24 months after surgery. Exclusion criteria were as follows: previous surgical or chemotherapeutic treatments, distant metastasis, absence of removal of cervical lymph nodes, and positive surgical margins.

The patients exhibited epidemiological profiles and classic prognoses of patients with OCSCC, such as: mean age of 63.7 ± 11.0 yr, including 54 men (69.3%) and 24 women (30.7%), with male sex - being a risk factor for recurrence (OR = 9,095, CI = 0.95–87.10, Table 3). The smoking status was 14 non-smokers (17.9%), 23 former smokers (29.5%), and 41 current smokers (52.6%). Alcohol use was 17 non-drinkers (21.8%), 27 former drinkers (34.6%), and 34 current drinkers (43.6%), with current alcohol consumption being identified as a risk factor for death by cancer (OR = 2.844, CI = 1.00–8.01; Table 3), as well as a risk factor for worse overall survival (HR = 1.804, CI = 0.80–4.06). Epidemiological and prognostic characteristics are summarized in Table 4.

**Table 4:** Characteristics of patients which oral squamous cell carcinoma.

Characteristic	All patients (n = 78)	Recurrence			Death		
		No (n = 63)	Yes (n = 15)	P-	No (n = 49)	Yes (n = 29)	P-
**Age**	63.7 (± 11.0)*						
≤ 63 years	41 (52.6)	29 (46.0)	12 (80.0)	***0.017***	22 (44.9)	19 (65.5)	0.078
> 63 years	37 (47.4)	34 (54.0)	3 (20.0)		27 (55.1)	10 (34.5)	
**Sex**							
Female	24 (30.7)	23 (36.5)	1 (6.7)	***0.020***	19 (38.8)	5 (17.2)	***0.039***
Male	54 (69.7)	40 (63.5)	14 (93.3)		30 (61.2)	24 (82.8)	
**Smoking status**							
Never	14 (17.9)	14 (22.2)	0 (0.0)	0.121	12 (24.5)	2 (6.9)	0.051
Former	23 (29.5)	17 (27.0)	6 (40.0)		16 (32.7)	7 (24.1)	
Current	41 (52.6)	32 (50.8)	9 (60.0)		21 (42.9)	20 (69.0)	
**History of tobacco consumption**							
No^1^	14 (17.9)	14 (22.2)	0 (0.0)	***0.037***	12 (24.5)	2 (6.9)	***0.045***
Yes^2^	64 (82.1)	49 (77.8)	15 (100.0)		37 (75.5)	27 (93.1)	
**Current tobacco consumption**							
No^3^	37 (47.4)	31 (49.2)	6 (40.0)	0.521	28 (57.1)	9 (31.0)	***0.022***
Yes^4^	41 (52.6)	32 (50.2)	9 (60.0)		21 (42.9)	20 (69.0)	
**Alcohol use**							
Never	17 (21.8)	17 (27.0)	0 (0.0)	***0.042***	13 (26.5)	4 (13.4)	***0.040***
Former	27 (34.6)	22 (34.9)	5 (33.3)		20 (40.8)	7 (24.1)	
Current	34 (43.6)	24 (38.1)	10 (66.7)		16 (32.7)	18 (62.1)	
**History of alcohol consumption**							
No^1^	17 (21.8)	17 (27.0)	0 (0.0)	***0.016***	13 (26.5)	4 (13.8)	0.151
Yes^2^	61 (78.2)	46 (73.0)	15 (100.0)		36 (73.5)	25 (86.2)	
**Current alcohol consumption**							
No^3^	44 (56.4)	39 (61.9)	5 (33.3)	***0.043***	33 (67.3)	11 (37.9)	***0.011***
Yes^4^	34 (43.6)	24 (38.1)	10 (66.7)		16 (32.7)	18 (62.1)	

^1^No; Never; ^2^Yes, Former/Current; ^3^No, Never/Former; ^4^Yes, Current; Data represented as absolute counts (%); ^*^Age, Mean (SD).

### Tissue microarrays

Tissue microarrays (TMA) were made using 10% buffered formalin-fixed sections embedded in paraffin of 78 primary oral cavity epidermoid carcinomas from patients surgically treated at the ICAVC, following the methodology described by Cajaiba *et al*.^54^. Two representative tumor areas were examined by two experienced pathologists from slides stained with hematoxylin and eosin. Two 1.5-mm diameter cylinders were drilled into each sample and reintroduced into paraffin receptor blocks using a tissue microarrayer (BEECHER INSTRUMENTS, Silver Spring, MD, USA). Sections were then removed from the TMA and mounted on microscopy slides. A pathologist checked the content of each spot. Spots bent or missing more than 70% of the tissue were excluded.

### Qualitative elemental characterization

For the qualitative elemental characterization, tumor tissue samples (mean thickness of 450 μm; mean density of 0.54 g/cm^3^) were removed from the TMA and subjected to dewaxing and rehydration processes with xylene, alcohol (the quality and integrity of these reagents were checked for purity in each batch used) and ultrapure water. They were then deposited in plastic support with Ultralene film (SPEX SAMPLEPREP, Metuchen, NJ, USA) and sent to the D09-XRF beamline equipment at the Brazilian Synchrotron Light Laboratory, Campinas, São Paulo, Brazil^55^, for analysis by micro X-Ray Fluorescence (µ-XRF). To obtain the spectra, a white beam with a power range of 4 to 24 keV and dimensions of 2 mm^2^ was used to excite the samples for 20 seconds. The fluorescent X rays emitted by the samples were detected using a high-resolution spectrometer, based on a Silicon Drift detector with an 8-μm thick beryllium window and an active area of 7 mm^2^. A 45-mm aluminum filter was used in the bundle and the sample holder, positioned at 21 mm and at a 45° angle to the detector. All measurements were performed at a temperature of 23 ± 2 °C and a pressure of 1 ATM.

Nine measurements were performed in a 3×3 matrix and were averaged to obtain the final spectrum used in the analyses. For the adjustment of the characteristic X-ray spectra, determination of the elements and their respective fluorescent intensities, a certified reference sample, Standard Reference Material 1577b “Bovine Liver” (NATIONAL INSTITUTE OF STANDARDS AND TECHNOLOGY, Gaithersburg, MD, USA), was subjected to the same experimental parameters described above. Analysis of the spectrum and determination of the characteristic peaks of each element was carried out using PYMCA 5.0.0^56^. Each spectrum was verified, the characteristic peaks of the elements identified, and then identification values were assigned as (0) for absence and (1) for the presence of the characteristic peak.

### Statistical analysis

Samples were categorized according to patients’ smoking habits: nonsmokers (never), past smokers (former) and current smokers (current). They were categorized by the history of tobacco consumption (No: never; Yes: former/current) and by current tobacco consumption (No: never/former; Yes: current). The chi square and Fisher exact tests were used for association analysis (p < 0.05 considered significant). Multivariable logistic regression was used to obtain OR and CI (CI ≥ 95%). Overall survival was calculated as the number of months between surgery and death for each patient or the last appointment for surviving patients. To calculate recurrence-free survival, the endpoint was the date of disease recurrence. The Kaplan–Meier model was used for survival analysis, using the Wilcoxon p-value and the Cox proportional hazards to adjust p-values, and to HR and CI (CI ≥ 95%). The values of OR and HR were adjusted for lymph node status (TNM). All analyses were performed using SPSS version 20 (IBM Corp., Armonk, NY, USA).

### Ethics declarations

The GENCAPO project was approved by National Research Ethics Commission (CONEP) [Technical advice: 128/2012; CONEP: 16491]. This study was approved by the Committees of Ethics Research of the Federal University of Espírito Santo [CAAE: 49091515.9.0000.5060] and the Arnaldo Vieira de Carvalho Cancer Institute [CAAE: 49091515.9.3002.5471]. The study was carried out in accordance with all relevant guidelines. As this was a retrospective study, the requirement for informed consent was waived.
